# Shared genetic correlation and causal association between major depressive disorder and breast cancer

**DOI:** 10.1186/s12916-023-02905-8

**Published:** 2023-06-06

**Authors:** Kazutaka Ohi

**Affiliations:** 1 Department of Psychiatry, Gifu University Graduate School of Medicine, 1-1 Yanagido, Gifu, 501-1194 Japan; 2 Department of General Internal Medicine, Kanazawa Medical University, Ishikawa, Japan

**Keywords:** Major depressive disorder, Breast cancer, Genetic correlation, Causal association, Inflammation

## Background

Major depressive disorder (MDD) and breast cancer (BC) are two distinct health conditions that can be linked in several ways. It is well established that cancer patients have a higher risk of developing MDD than the general population [1]. A diagnosis of BC can be a life-changing event, and treatments, such as surgery, chemotherapy, and radiation therapy, can be physically and emotionally stressful. This may lead to symptoms of anger, fear, anxiety, and depression with regard to the disease and treatment process. Furthermore, some of the side effects of BC treatment, such as pain, fatigue, and changes in hormone levels, can contribute to the development of MDD. Thus, BC patients are more likely to experience depressive symptoms after diagnosis and treatment than individuals without BC [2]. Moreover, several studies have found that individuals with MDD have a higher risk of developing BC than those without MDD [3–5], though this population-based evidence remains inconsistent among studies. Including the recent large-scale longitudinal follow-up study from the UK Biobank by Wu et al. (2023), individuals with MDD reportedly have a 1.1- to 2.5-fold increased risk of developing BC compared to those without MDD. Although there is evidence to suggest that BC may increase the risk of developing MDD and vice versa, further research is required to fully understand the relationship. Recently, Wu et al. (2023) have reported the phenotypic as well as genetic relationships between MDD and BC [5].

## Involvement of genetic factors in the pathogenesis of MDD and BC

Several studies have suggested that the relationship may be due to the impacts of stress and chronic inflammation on the immune system, which might increase the risk of both MDD and BC. Psychosocial stressors in cancer as well as depression itself promote inflammation and oxidative/nitrosative stress, decreased immunosurveillance, and dysfunctional activation of the autonomic nervous system and the hypothalamic‒pituitary‒adrenal (HPA) axis.

Genetic factors play a role in the pathogenesis of MDD and BC, with heritability estimates of 37% [6] and 31% [7], respectively. To date, large-scale genome-wide association studies (GWASs) of MDD [8] and BC [9] have identified over 100 and 150 genome-wide significant loci related to these conditions, respectively. Furthermore, genetic correlations between risk of MDD and female reproductive phenotypes such as age at menarche and menopause (*r*_*g*_≈-0.12), which are well-established risk factors for BC, have been demonstrated [8]. Wu et al. (2023) indicated that common genetic factors play a role in the pathogenesis of both MDD and BC (*r*_*g*_ = 0.08) regardless of subtype based on the status of estrogen receptor (ER) (ER + , *r*_*g*_ = 0.06; ER–, *r*_*g*_ = 0.08) [5]. In addition, Wu et al. (2023) identified shared specific local genomic regions, such as at 6p22.1, shared specific loci at the individual variant level, such as rs2403907 at 21q21.1, shared genes, such as *FLOT1* and *HLA-S* at 6p21.33, enriched in specific tissues including brain, blood, uterus, colon, artery and heart, and shared genetic functions, such as immune, inflammatory, and stress responses [5], which may be involved in the development of both MDD and BC. These findings support that MDD and BC may be linked by shared biology.

## Causal association between MDD and BC

There is evidence for an association between MDD and BC based on observational studies and genetic studies, though the direct causal relationship between the two conditions is not yet fully understood. Mendelian randomization (MR) analysis is a method that uses genetic variants associated with exposure as instrumental variables to establish causal relationships between exposures and outcomes. MR analysis depends on the natural randomized assortment of genetic variants, making it an analog for randomized controlled trials, and it can be used to infer causality in large-scale GWAS datasets. Using MR analysis, bidirectional causal associations between MDD and BC were investigated in two independent studies [5, 10], including the recent study by Wu et al. (2023). Both studies detected the causal effect of MDD on BC (OR = 1.09–1.12), but the causal effect of BC on MDD was not detected (OR = 1.00–1.01) [5, 10]. MDD was associated with an increased risk of BC. In subgroup analysis divided by ER status, the effect of depression on ER– BC (OR = 1.10–1.12) was slightly stronger than that of depression on ER + BC (OR = 1.07–1.08). Similarly, there was no evidence of reverse causality associated with ER status.

## Conclusions

In light of the new findings of Wu et al. (2023) [5], this article discusses the association between MDD and BC, the extent to which these conditions coexist in observation studies, and the extent to which genetic factors play a role in the pathogenesis of both conditions. BC and its treatment can cause physical and emotional stress, leading to MDD development. Conversely, MDD is a risk factor for BC development. There is evidence to suggest that MDD and BC have shared genetic influences, with large-scale GWASs identifying multiple significant loci related to MDD and BC and these genetic correlations. Additionally, the causal relationship between MDD and BC is discussed herein. The potential causal effect of MDD on BC is suggested, but reverse causality has not been identified. Overall, environmental factors, including physical and emotional stress associated with the diagnosis and treatment of BC, may contribute to the development of MDD in BC patients, and there may be a biological link between MDD and BC, with chronic inflammation and other biological changes associated with MDD potentially increasing risk of BC. These findings highlight the importance of physicians addressing the physical and emotional needs of patients with these conditions and the importance of mental health in the prevention and treatment of BC.

## Data Availability

Not applicable.
